# Supplementary material for: miR-145-5p Targets Sp1 in Non-Small Cell Lung Cancer Cells and Links to BMI1 Induced Pemetrexed Resistance and Epithelial–Mesenchymal Transition
Source: Int J Mol Sci. 2022 Dec 5;23(23):15352. doi: 10.3390/ijms232315352 (PMC9741456; doi:10.3390/ijms232315352)
Supplement: Supplementary file 1 [file ijms-23-15352-s001.zip › ijms-2054920-supplementary.pdf]

International Journal of
*Molecular Sciences*

MDPI

# Sp1 is targeted by miR-145-5p in non-small cell lung cancer cells and participates BMI1 induced pemetrexed resistance and epithelial-mesenchymal transition

**Wen-Wei Chang [1, 2#], Bing-Yen Wang [3,4#], Shih-Hong Chen [1], Peng-Ju Chien [1], Gwo-Tarng Sheu [5] and Ching-Hsiung Lin [6,7,8,9*]**

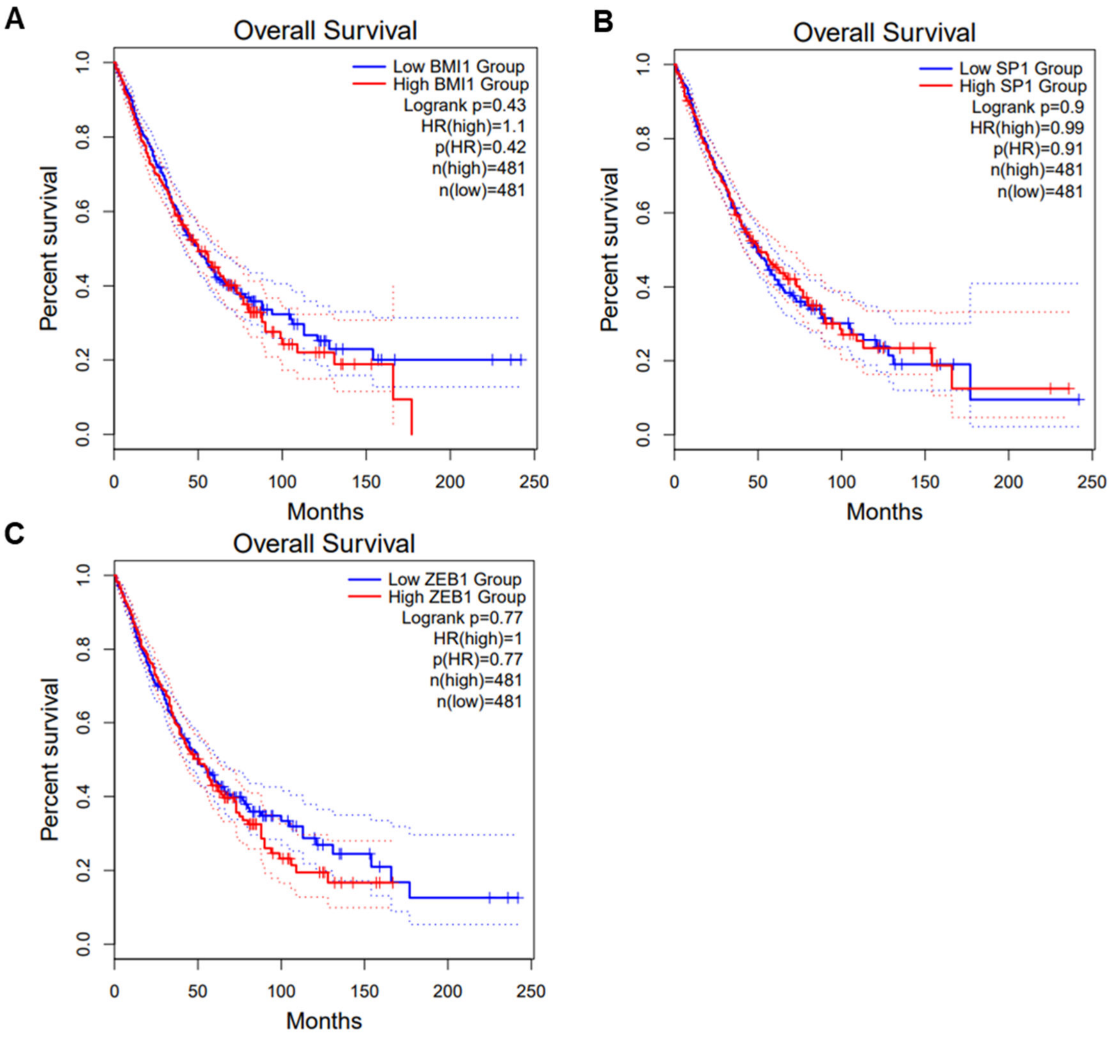


**Figure S1. Survival analysis of BMI1, SP1, or ZEB1 in NSCLC patients.** Survival analysis based on single gene of BMI1, SP1, or ZEB1 in NSCLC subjects of TCGA database was analyzed using GEPIA2 webtools using median expression level as the cut-off. The statistical analysis was performed by a log rank test.

**Table S1. The information of antibodies used in this study.**

| ***Product*** | ***Source*** | ***Catalogue No.*** |
|---|---|---|
| **Primary antibody:** | | |
| anti-BMI1 | Cell Signaling Technology, Inc. | 6964s |
| anti-E-Cadherin | Santa Cruz Biotechnology, Inc | sc-21791 |
| anti-N-Cadherin | Santa Cruz Biotechnology, Inc | sc-59987 |
| anti-SNAIL1 | Cell Signaling Technology, Inc. | 3879s |
| anti-ZEB1 | GeneTex International Corporation | GTX105278 |
| anti-GAPDH | GeneTex International Corporation | GTX100118 |
| Anti-β-actin | Sigma-Aldrich | A5441 |
| **Secondary antibodies:** | | |
| anti-rabbit IgG-HRP | GeneTex International Corporation | GTX221666-01 |
| anti-mouse IgG-HRP | GeneTex International Corporation | GTX221667-01 |

**Table S2. Primer Sequences for the construction of Sp1 3’-UTR reporter vectors.**

| *Primer name* | *Sequences (5’ to 3’)* |
|---|---|
| WT-F-EcoRI | GAATTCTGTGGGATAAAGATTATGATTAGGG |
| WT-R-XbaI | TCTAGAGCTGCGACCTTTCTTTCATC |
| Mut-F | AGCTGCAGATCTTTGCTAG |
| Mut-R | ACAATGTGAAGATATTAACAATACAAAATATATTTTG |
